# RNA-seq dataset of female germline stem cells treated with sodium lactate

**DOI:** 10.1016/j.dib.2026.113348

**Published:** 2026-10-14

**Authors:** Yanyi Guo, Bo Xu, Jeffrey Jianan Gu, Yanlin Ma, Xiaopeng Hu, Ji Wu

**Affiliations:** a Key Laboratory for the Genetics of Developmental & Neuropsychiatric Disorders (Ministry of Education), Bio-X Institutes, Shanghai Jiao Tong University, Shanghai, 200240, China; b Key Laboratory of Fertility Preservation and Maintenance of Ministry of Education, School of Basic Medical Sciences, Ningxia Medical University, Yinchuan, 750004, China; c Hainan Provincial Key Laboratory for Human Reproductive Medicine and Genetic Research, Hainan Provincial Clinical Research Center for Thalassemia, Key Laboratory of Reproductive Health Diseases Research and Translation (Hainan Medical University), Ministry of Education, the First Affiliated Hospital of Hainan Medical university, Hainan Medical niversity, Haikou, Hainan, 571101, China; d Emory University Rollins School of Public Health, GA, USA

**Keywords:** Lactylation, Differentiation, Meiosis

## Abstract

Female germline stem cells(FGSC) are a type of adult stem cell found in the ovaries of animals which possess the ability to self-renew in vitro and differentiate into mature oocytes. Lactylation is a widespread post-translational modification of proteins that plays a crucial role in biological processes such as cell fate regulation and cell signaling, and has been shown to significantly influence processes such as immunity and development. However, the effects of altered lactylation on the development of female germline stem cells remain unclear. Therefore, we used sodium lactate treatment to modify the lactylation status of mouse-derived female germline stem Y

cells(Supplementary Fig. 1) and employed RNA-seq technology to detect and analyze changes in lactylation-related genes and development-related genes. Through data cleaning of sequencing reads, genome alignment, and statistical testing, a total of 14,376 expressed genes were identified, of which 1,827 were significantly upregulated and 417 were significantly downregulated. We investigated the effects of altered lactic acid modification on the transcriptomic expression of female germline stem cells using GSEA enrichment analysis and mRNA alternative splicing analysis. This dataset will help us understand the effects of lactylation on processes such as proliferation and differentiation in female germline stem cells, providing a reference for research into the development of female germ cells.

Specifications Table

**Table:** 

Subject	Biology
Specific subject area	Reproductive Physiology, Epigenetic
Type of data	Table, Image, Chart, Graph, Figure etc. Raw, Analyzed, Filtered, Processed etc.
Data collection	FGSCs were treated with 40 mM sodium lactate for 48 hours, collected as the treatment group, and compared with the control group. Raw transcriptomic data were obtained via Illumina NovaSeq 6000 platform sequencing. Adapters were removed and reads filtered using Cutadapt. Alignment was performed with Fastqc and HISAT2 against the mm10 reference genome. Annotation was conducted with StringTie against ENSEMBL10. Differential gene analysis was performed using edgeR.
Data source location	Institution: Bio-X institutes, Shanghai Jiao Tong University City: Shanghai Country: China.
Data accessibility	Repository name: NCBI-SRA Data identification number: PRJNA1482551 Direct URL to data: https://www.ncbi.nlm.nih.gov/bioproject/?term=PRJNA1482551
Related research article	None

## 1 Value of the Data

• This dataset illustrates changes in mRNA expression and splicing in female germline stem cells following treatment with sodium lactate.
• This dataset presents the overall changes in the transcriptome of female germline stem cells during the process of lactylation modification, providing a reference for studying the relationship between lactylation modification and germ cell development.
• This dataset offers potential gene targets for the study of transcriptomic alterations in female germline stem cells subjected to sodium lactate induction.

## 2 Background

Female germline stem cells are a type of adult stem cell capable of self-renewal in vitro and differentiation into mature oocytes. First isolated and identified in mice [1], subsequent research has successfully isolated and characterized these cells in pigs [2], monkeys [3], sheep [4], and humans [5]. Their cellular functions have been validated through in vitro induction and in vivo injection. Lactylation is a post-translational modification of proteins widely present in animal organisms. First discovered during studies of lipopolysaccharide-stimulated macrophages [6], research has demonstrated its close association with diseases such as immune disorders and cancer, as well as its regulatory role in germ cell development [7]. Therefore, investigating changes in gene expression levels of FGSCs following sodium lactate treatment can provide valuable insights into the relationship between lactylation and FGSC development, potentially identifying key gene targets.

## 3 Data Description

Based on the proliferation and differentiation profiles of female germline stem cells cultured for 48 hours with different sodium lactate concentrations, 40 mM sodium lactate for 48 hours was chosen as the treatment condition for subsequent experiments(Supplementary Fig 2).We obtained raw data through transcriptomic sequencing and generated reliable sequencing reads through de-tagging, data filtering, and quality control (Fig 1, Fig 2). We assessed sequencing quality and intra-sample reproducibility by analyzing the distribution of nucleotide frequencies and their correlations across samples, and used principal component analysis to visualize sample clustering (Fig 3). By aligning the sequencing data with the reference genome, we identified a total of 14,376 expressed genes, of which 1,827 were significantly upregulated and 417 were significantly downregulated (Fig 4A, B). We analyzed the functions and characteristics of the differentially expressed genes using GO and KEGG (Fig 4C, D). We analyzed changes in genes related to female germ cell development following sodium lactate treatment (Fig 5). Furthermore, we employed GSEA cluster analysis to examine gene changes in specific developmental processes (Fig 6) and analyzed alternative splicing patterns in mRNA during these processes (Supplementary Information). Through transcriptomic sequencing and subsequent analyses, we have provided a data reference for studying lactation and germ cell development, and offered insights for further research into the effects of lactation on gene expression.

**Fig 1:**
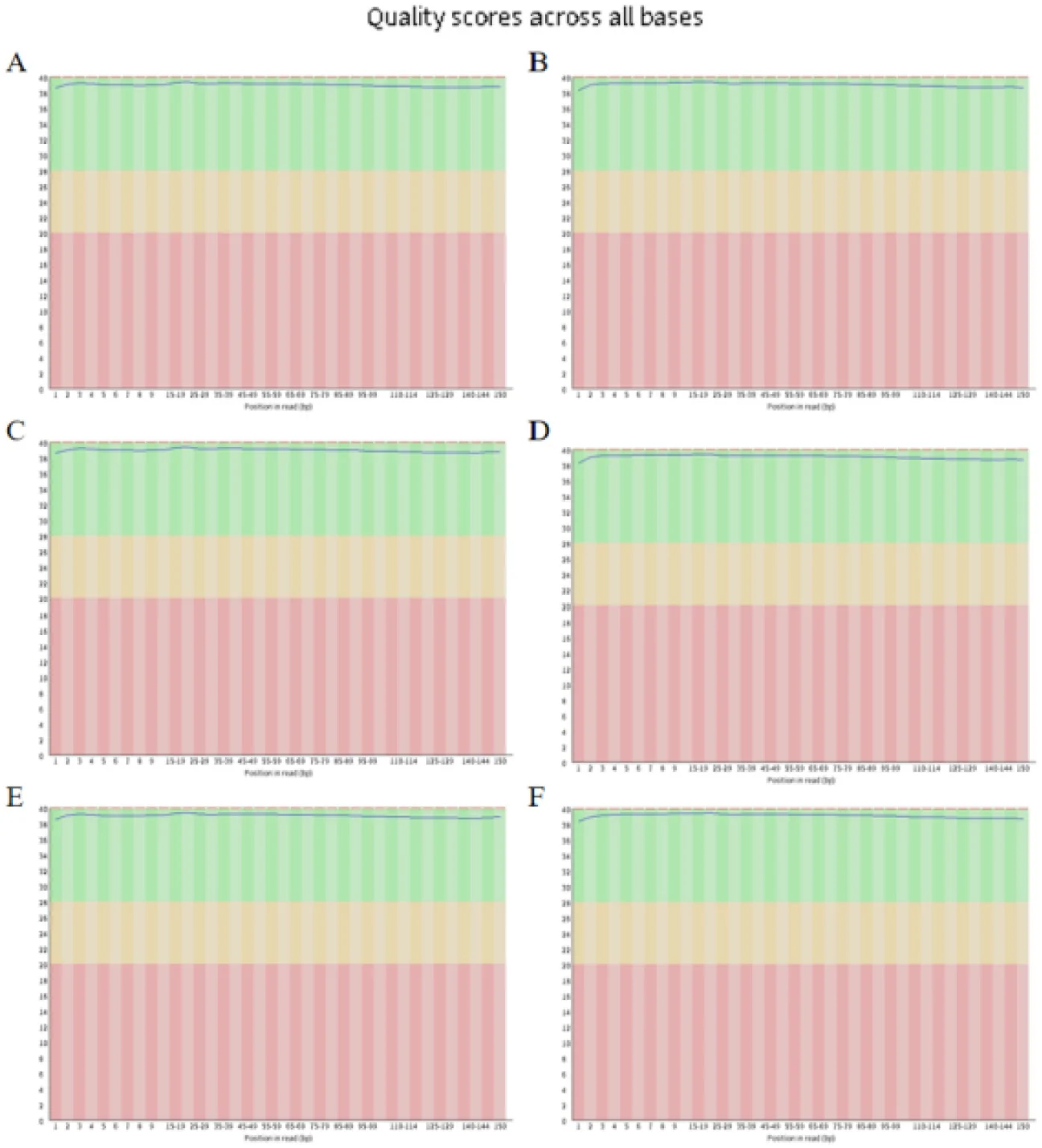
FGSC dual-end sequencing single-base quality map for the natural control group (A,B) NC1 (C,D) NC2 (E,F) NC3.

**Fig 2:**
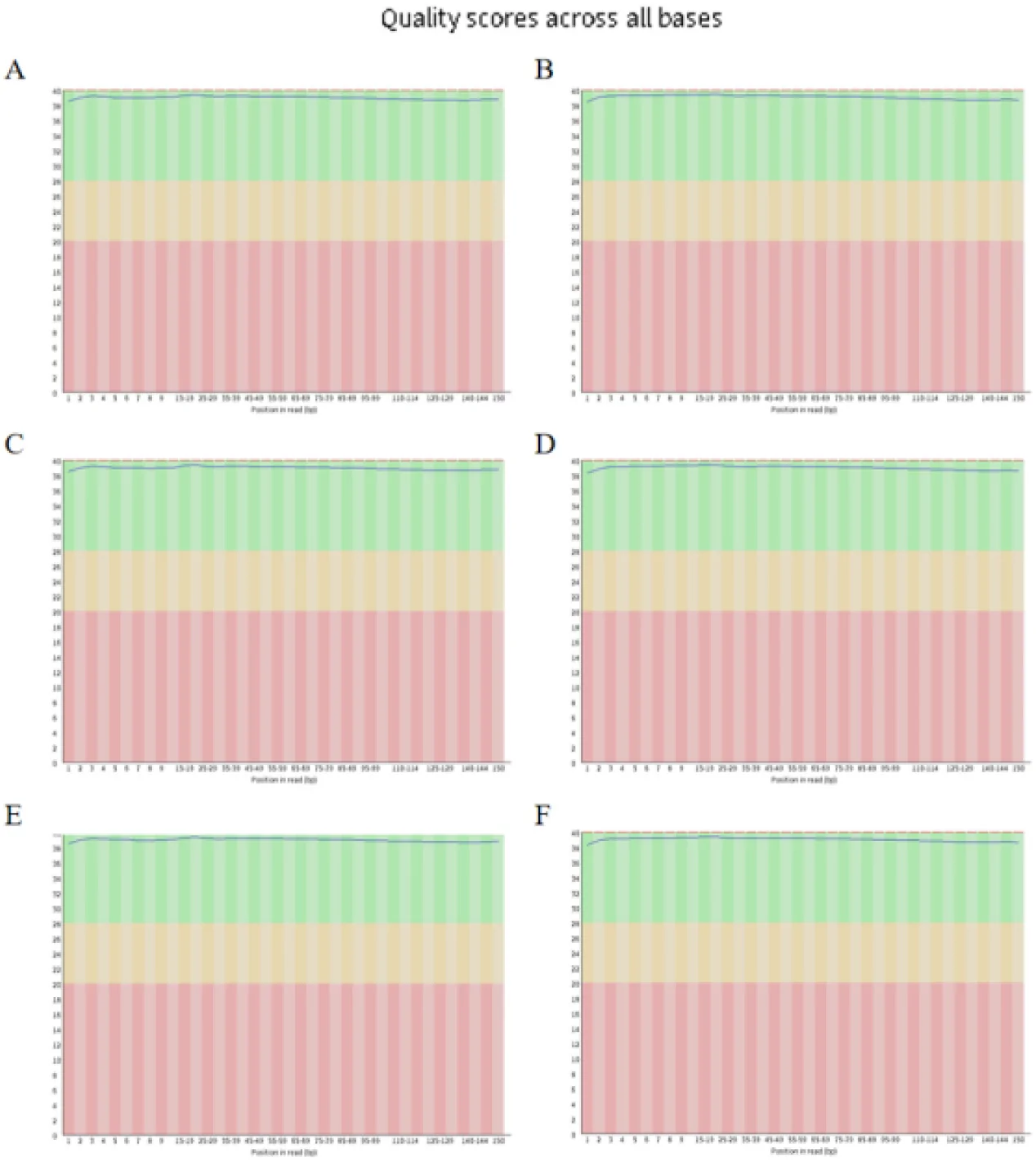
FGSC dual-end sequencing single-base quality map for the experimental group (A,B) EX1 (C,D) EX2 (E,F) EX3.

**Fig 3:**
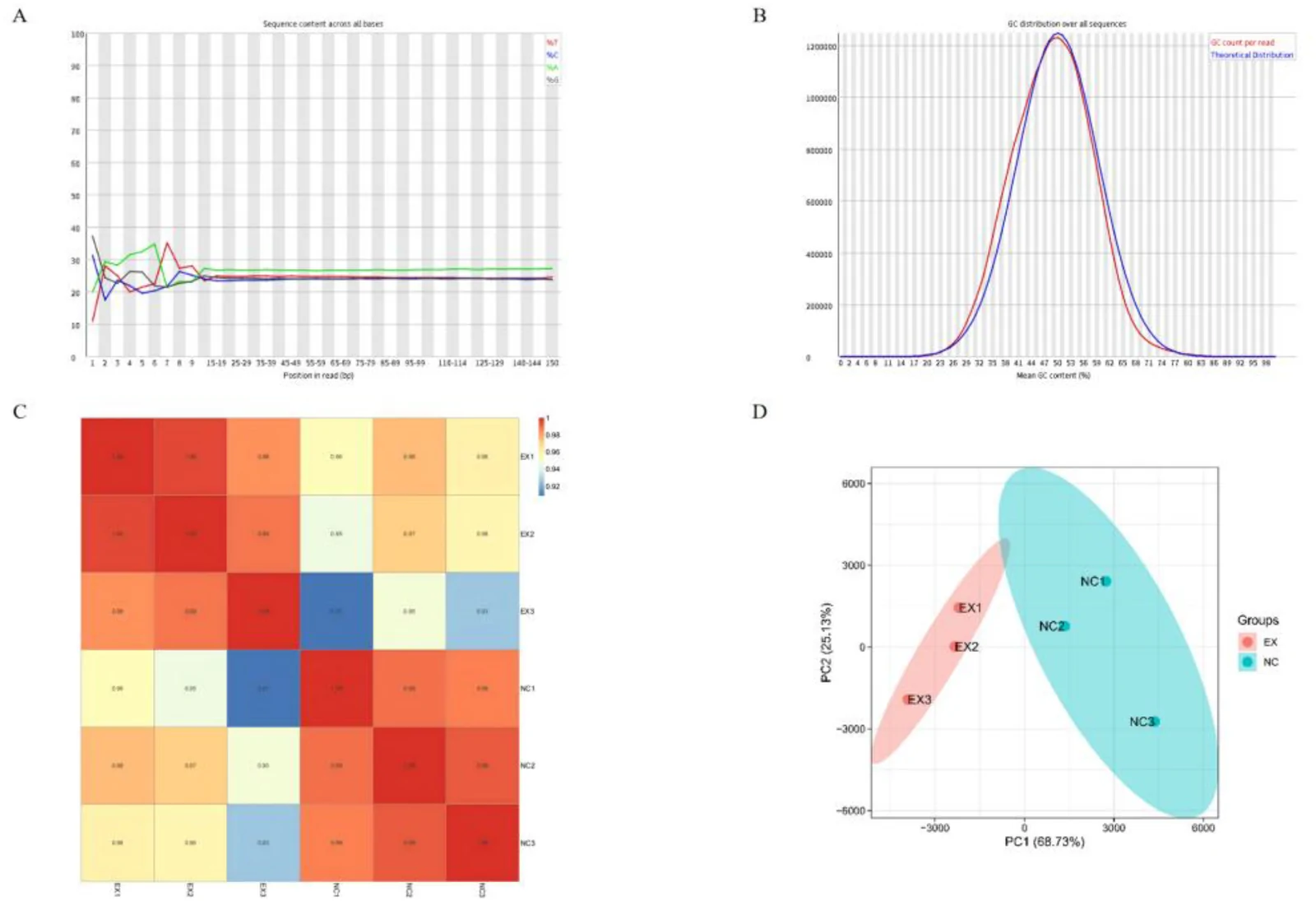
(A) Proportion of the four nucleotides A/T/C/G in sequencing data (B) Comparison of GC content in sequencing results versus standard normal distribution (C) Inter-sample correlation data calculated using Pearson's method (D) Principal component analysis results for FGSC control group versus experimental group.

**Fig 4:**
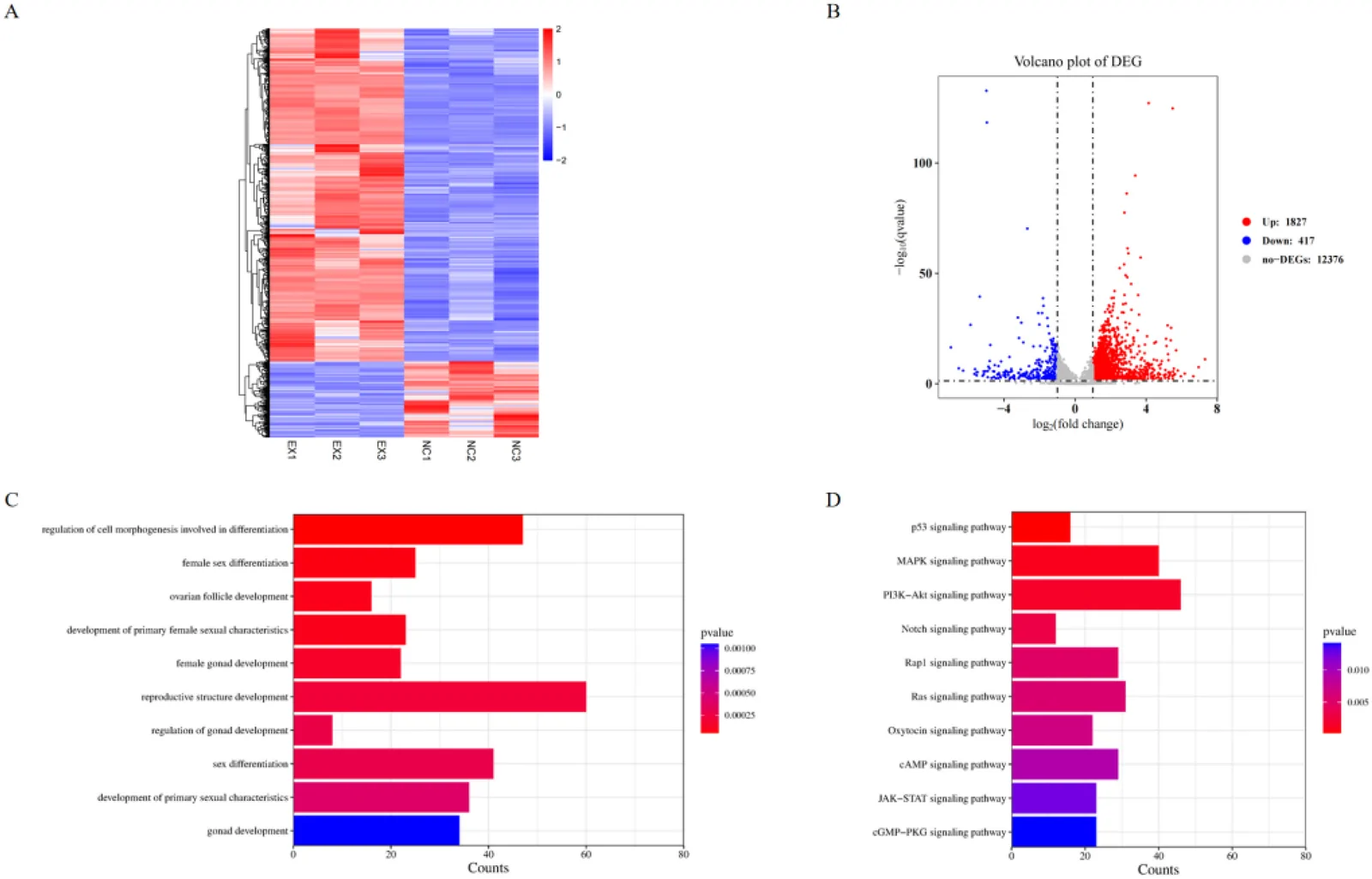
(A) Hierarchical clustering heatmap displays expression patterns of differentially regulated genes across samples, with gene expression values transformed using the Z-score method. (B) Volcano plot of differentially regulated genes between the FGSC control group and experimental group. The X-axis shows log2-transformed fold change values for all genes, while the Y-axis displays -log10-transformed q-values. Red dots indicate significantly upregulated genes (q-value < 0.05, log2FC > 1), while blue dots denote significantly downregulated genes (q-value < 0.05, log2FC < -1). The vertical dashed line represents |log2FC| = 1, and the horizontal dashed line indicates q-value = 0.05. (C, D) Results of GO and KEGG enrichment analyses. The horizontal axis represents the number of differentially expressed genes. Colors indicate p-value values, with red indicating lower p-values and higher significance.

**Fig 5:**
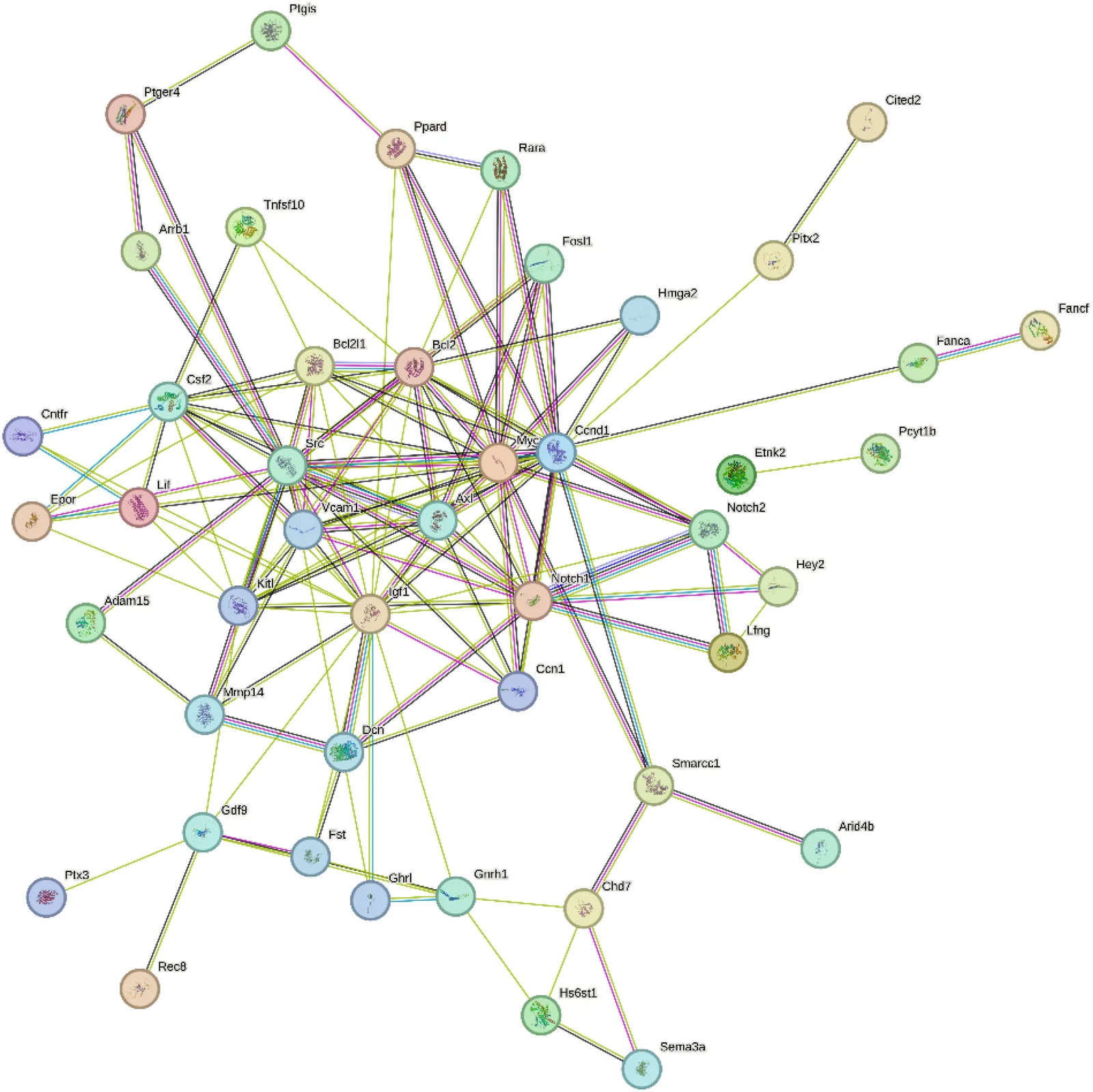
Differential protein interaction network between the natural control group and the experimental group, highlighting selected genes associated with female germ cell development.

**Fig 6:**
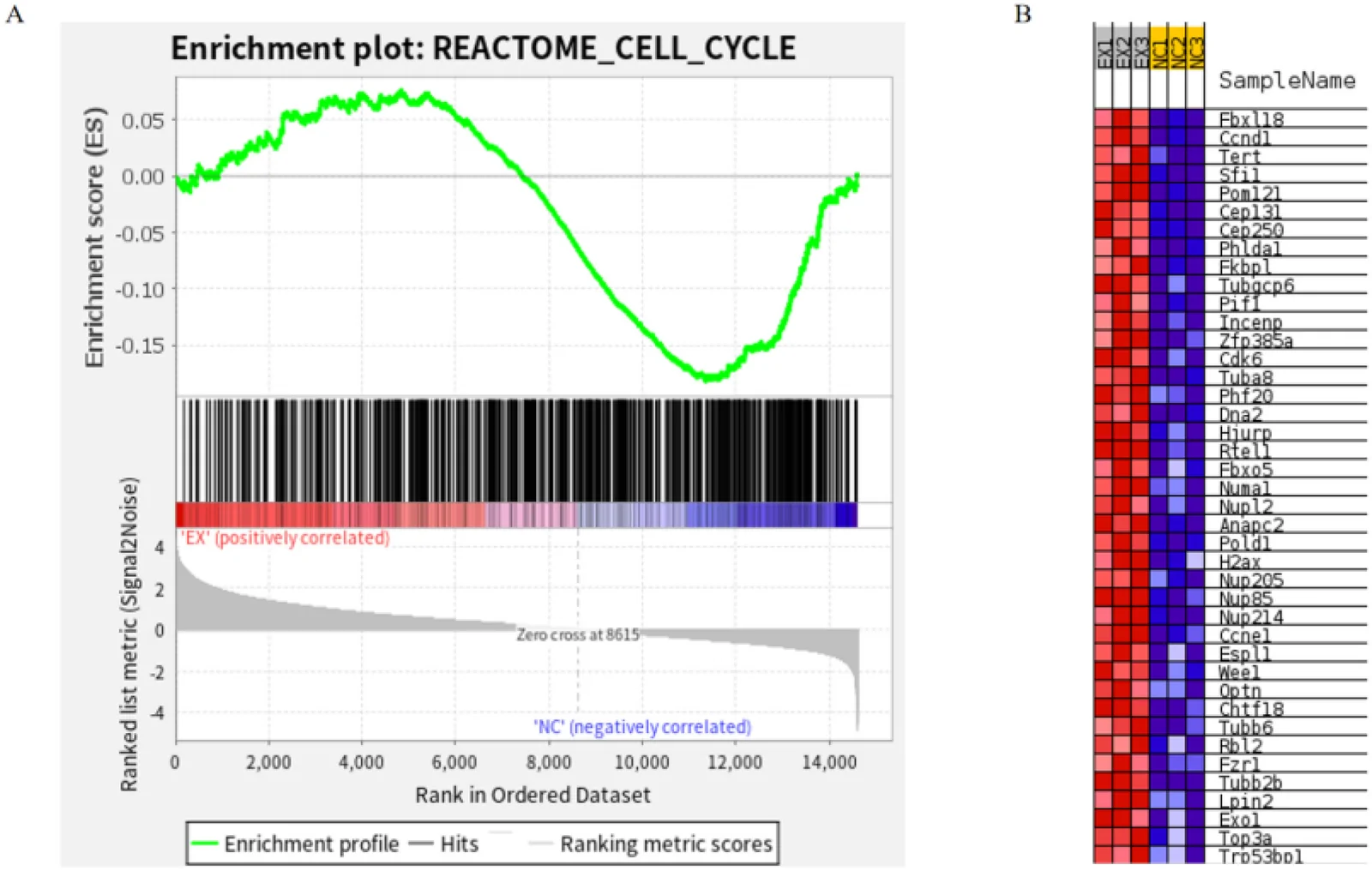
Representative RNA-Seq GSEA analysis results A: Changes in the enrichment score (ES) for this pathway B: Gene expression patterns for this pathway (red indicates high gene expression levels; blue indicates low gene expression levels).

## 4 Experimental Design, Materials and Methods

### 4.1 Sodium lactate treatment of FGSCs

FGSCs at passages P5–P10 were used in this study. Cells were seeded into individual wells of a 24‑well plate at a density of 1 × 10⁴ cells per well. The cells were divided into natural control group(NC) and experimental group(EX). The control group was cultured in standard FGSC medium(MEM-α, 10% FBS, 1 mM sodium pyruvate, 1 mM non-essential amino acids, 2 mM L-glutamine, 0.1 mM β-mercaptoethanol, 10 ng/mL LIF, 10 ng/mL EGF (mouse epidermal growth factor), 40 ng/mL GDNF (glial cell line-derived neurotrophic factor), 1 ng/mL bFGF (basic fibroblast growth factor) and 15 mg/L penicillin.) for 48 hours, while the experimental group was cultured in the same medium supplemented with 40 mM sodium lactate for an equal duration (48 hours). After incubation, cell samples were collected by trypsin digestion.

### 4.2 Sample preparation and sequencing

Total RNA was extracted using the Trizol method. RNA fragments bearing polyA tails were captured via oligo-dT magnetic beads. mRNA was denatured by heating, then incubated with reverse transcription mix to transcribe mRNA into cDNA. Sequencing adapters were ligated to the 3′ ends of DNA fragments. In vitro amplification and electrophoresis were performed to select DNA fragments of 300–400 bp for sequencing. Sequencing was conducted on the Illumina NovaSeq 6000 platform.

### 4.3 Data analysis

For the raw sequencing data, Cutadapt was used to remove adapters and filter out low-quality reads, followed by Fastqc, HISAT2 against the mm10 reference genome, and assembled the raw sequencing data using StringTie against the ENSEMBL10 annotation. Gene expression levels were analyzed and calculated, with edgeR used for differential gene analysis, clusterProfiler for GO/KEGG pathway analysis of differentially expressed genes, and rMATS for transcriptional splicing variation analysis.

## Limitations

In this dataset, due to cost constraints, we only examined the overall transcriptomic changes following treatment with 40 mM sodium lactate for 48 hours. Transcriptome sequencing was not performed for other treatment concentrations or time points.

## Ethics Statement

We have declared that we have read and follow the ethical requirements for publication in Data in Brief and confirm that the current work does not involve human subjects, animal experiments, or any data collected from social media platforms.

## CRediT Author Statement

**Yanyi Guo:** Formal analysis, Investigation, Methodology, Software, Validation, Visualization,Writing – original draft**, Bo Xu:** Resources, Supervision,Investigation, Validation, Visualization**, Jeffrey Jianan Gu:** Data curation, Investigation, Validation**, Yanning Ma:** Resources, Supervision, Writing – review & editing**, Xiaopeng Hu:** Conceptualization, Funding acquisition, Resources, Supervision, Writing – original draft, Writing – review & editing**, Ji Wu:** Project administration, Conceptualization, Funding acquisition, Resources, Supervision, Writing – review & editing.

## Data Availability

NCBI-SRARNA-seq data of Female Germline Stem Cell (Original data).

## Appendix Supplementary materials

Image, application 1
