# Supplementary material for: RNA-seq dataset of female germline stem cells treated with sodium lactate
Source: Data Brief. 2026 Oct 14;69:113348. doi: 10.1016/j.dib.2026.113348 (PMC13718652; doi:10.1016/j.dib.2026.113348)
Supplement: Supplementary file 1 [file mmc1.docx]

**Fig1** Changes in protein lactylation in female germline stem cells after treatment with different concentrations of sodium lactate(Con: control group; 5 mM, 10 mM, 20 mM, 40 mM, and 80 mM correspond to female germline stem cells treated with sodium lactate at the respective concentrations for 48 hours).

**Fig2 A:** Cell proliferation was assessed using the EdU assay. Statistical analysis of EdU‑positive cells was performed, and all data are expressed as mean ± SD from three biological replicates. **B:** Representative Western blots of Sycp1, Stra8, and Sycp3 (typical differentiation markers).
